# Reduced gravity promotes bacterially mediated anoxic hotspots in unsaturated porous media

**DOI:** 10.1038/s41598-020-65362-w

**Published:** 2020-05-25

**Authors:** Benedict Borer, Joaquin Jimenez-Martinez, Roman Stocker, Dani Or

**Affiliations:** 1 Department of Environmental Systems Science, ETHZ, Zürich, Switzerland; 2 Department of Water Resources and Drinking Water, EAWAG, Dübendorf, Switzerland; 3 Department of Civil, Environmental and Geomatic Engineering, ETHZ, Zürich, Switzerland

**Keywords:** Soil microbiology, Environmental sciences

## Abstract

Human endeavours into deep space exploration and the prospects of establishing colonies on nearby planets would invariably involve components of bioregenerative life support for food production, cabin atmosphere renewal, and waste recycling. Growing plants and their microbiomes in porous media under different gravitational fields may present new challenges due to effects of liquid distribution on gaseous exchange with roots and microorganisms. We provide the first direct evidence that capillary driven liquid reconfiguration in porous media under reduced gravity conditions reduces oxygen diffusion pathways and enhances anoxic conditions within bacterial hotspots. Parabolic flight experiments using model porous media inoculated with aerobic and facultative anaerobic bacteria reveal the systematic enhancement of anoxic conditions during the reduced gravity periods in the presence but not in the absence of bacterial activity. The promotion of anoxic conditions under reduced gravity may lead to higher nitrous oxide and methane emissions relative to Earth conditions, on the other hand, anoxic conditions could be beneficial for perchlorate bioremediation of Martian soil. The results highlight changes in soil bacterial microhabitats under reduced gravity and the challenges of managing bioregenerative life support systems in space.

## Introduction

Deep space exploration and the establishment of human colonies on the moon and nearby planets are likely to involve components of plant-based life support systems for food production, cabin atmosphere renewal and waste/water recycling^1–3^. Life support systems are commonly categorized as physico-chemical (PCLSS) or biological regenerative life support systems (BRLSS)^4–6^. Although PCLSS have benefits in terms of controllability and reliability, the elaborate hardware requirements and limited renewability^2^ leave ample room for consideration of BRLSS, which offers additional services, such as supplementing fresh food for the crew. The challenge with BRLSS is their limited predictability and untested stability for long missions. A key component of any BRLSS is the use of plants for nutrient recycling: consumption of carbon dioxide, production of oxygen, recycling of nitrogen from urine, and food production^4^.

The influence of reduced gravity on plant growth and reproductive capability has been investigated aboard various platforms in the past 40 years starting from the Salyut space station, the MIR station, Space Shuttle missions, and most recently aboard the International Space Station (ISS)^7^. Most of the growth systems tested relied on some form of porous medium acting as “soil” to provide nutrient supply and support matrix for the root zone^8–10^. However, the distribution of water in the unsaturated medium that governs nutrient and gas supply to plant roots is considerably different under reduced gravity in space compared to that under Earth’s gravity. In reduced gravity, capillary forces become dominant and result in a more uniform distribution of the aqueous phase within the porous medium relative to conditions on Earth where the aqueous phase is stratified with depth^11,12^. Due to the approximately thousand-fold lower diffusivity of gases in water relative to air, a more homogeneous distribution of the aqueous phase poses a barrier to gaseous diffusion within the porous matrix^13^. This creates conditions that can favor local anoxic hotspots due to root and microbial respiration, with profound consequences for plant and microbial activity and ultimately the functioning of the BRLSS.

The influence of reduced gravity on the onset of anoxia in porous media has been investigated using enzyme assays for anoxic stress response aboard both the MIR station and the Space Shuttle^14^, as well as direct measurements with oxygen sensors during parabolic flights^15^. These experiments demonstrated a reduction in oxygen within the root zone that has been attributed primarily to root respiration. However, such attribution ignores the role of microbial processes in the rhizosphere, often a prime microbial hotspot of activity compared to the surroundings^16^ and where microbial respiration could affect oxygen dynamics. Root zone hypoxia is detrimental for most plant species^17^, it stunts plant growth and could alter the composition of the rhizosphere microbial community^18^ favouring anaerobic metabolic activity (denitrification, methanogenesis) and associated greenhouse gas production^19^. The study of reduced gravity effects on anoxic bacterial hotspots could have beneficial applications (other than preventing detrimental effects on plant growth and greenhouse gas emissions), such as enhancing perchlorate remediation of Mars soil using anaerobic bacteria prior to agricultural, construction and other use^20,21^.

In this study, we provide direct evidence that bacteria contribute to the creation of anoxic hotspots in unsaturated porous media in reduced gravity, and that this effect is due to the homogenized distribution of the aqueous phase that results in the blocking of previously gas-filled pores. We use oxygen measurements with optodes in artificial pore networks inoculated with a synthetic bacterial community to directly visualize and quantify oxygen distribution and consumption in relation to the distribution of the aqueous phase in different gravity conditions during a parabolic flight. An advantage of parabolic flights was the ability to investigate anoxic conditions at varying gravitational accelerations, including zero gravity (0 *g*), Lunar gravity (0.16 *g*), Martian gravity (0.38 *g*) and hyper gravity (1.8 *g*). Our experiments demonstrate that, even during the short periods of zero gravity (22 s) of a parabolic flight, high-density bacterial colonies can render small volumes of a porous medium anoxic. We observed a consistent decrease in oxygen content in bacteria-loaded pore networks compared to sterile pore networks, with the magnitude of the effect varying with the strength of the gravitational acceleration.

## Results

**Figure 1:**
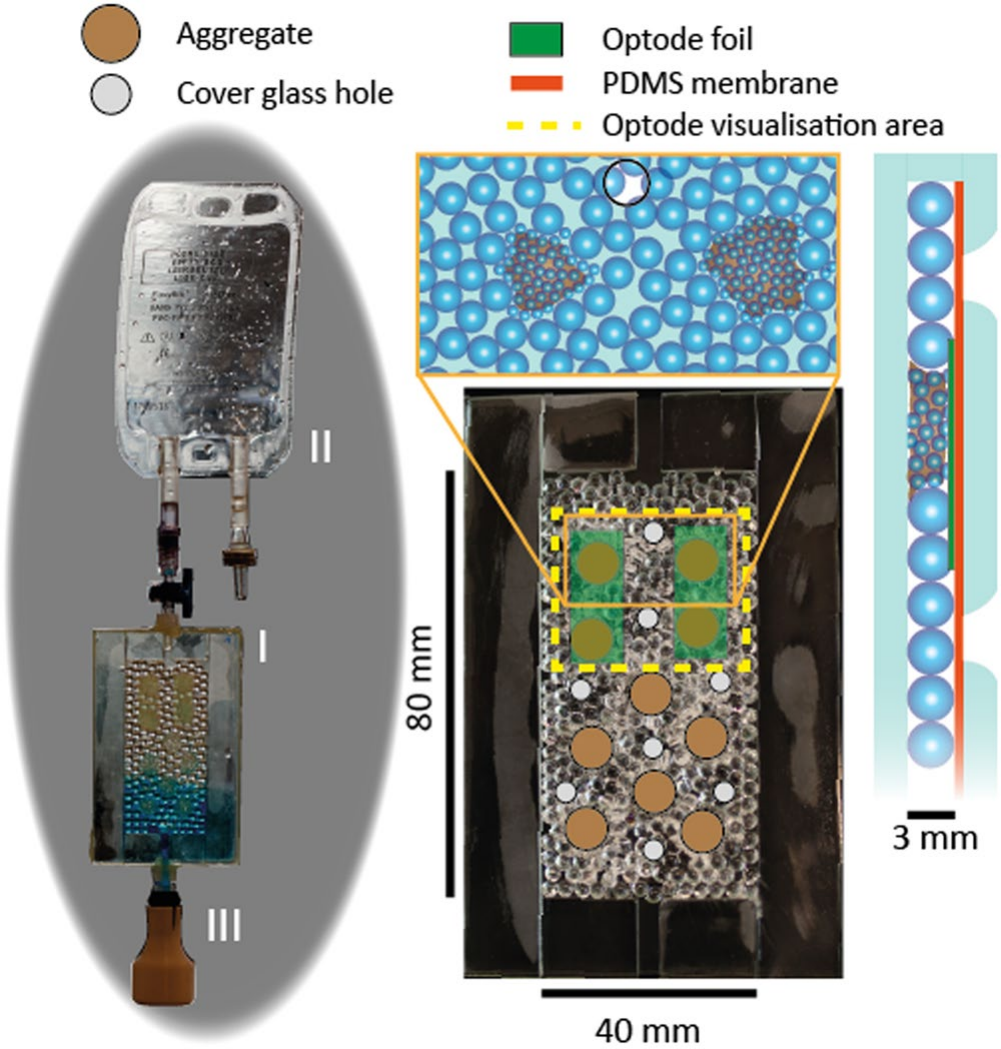
Fully assembled glass pore network and conceptual image. Assembled glass pore network (I), gas reservoir in the form of an IV bag (II) and liquid reservoir (III) enclosed in a flask for protection. Pore networks were formed by a sintered layer of 3-mm glass beads with the inclusion of 10 groups of 1-mm glass beads forming aggregates. Carbon in the form of solidified agar and bacterial cells were inoculated directly into the aggregates. The glass cover included aeration holes, with a polydimethylsiloxane (PDMS) membrane to prevent leakage of liquids. Optode images were taken from the upper four aggregates using optode foils placed onto the bead network.

Experiments were performed as part of the 3^rd^ Swiss Parabolic Flight campaign, which consisted of two flights and a total of 32 parabolas (14 zero-gravity parabolas, one Lunar gravity and one Martian gravity parabola per flight). Porous microcosms were fabricated to mimic the salient physical properties of unsaturated soils (Fig. 1; Methods). The primary porous matrix consisted of a sintered monolayer of large diameter glass beads in which pores remained mostly unsaturated (i.e., filled with air) during Earth gravity conditions. Embedded within this matrix, we placed inclusions consisting of small diameter glass beads mimicking soil aggregates, which were saturated (i.e., filled with liquid) even under Earth gravity conditions due to the dominance of capillary forces in their small pores. To ensure sufficient oxygen resupply during Earth gravity conditions, holes were drilled in the cover glass of the porous network device and covered with a polydimethylsiloxane (PDMS) membrane to permit gas diffusion whilst preventing liquid leakage. A carbon source was incorporated in each aggregate in the form of nutrient agar. The oxygen distribution was visualized using optodes made of oxygen-sensitive films attached to the cover glass so as to span the upper four aggregates. Experiments were conducted under different gravity conditions (SI Fig. 1) with both aerobic and facultative anaerobic bacterial species and compared to control experiments in sterile porous networks.

**Figure 2:**
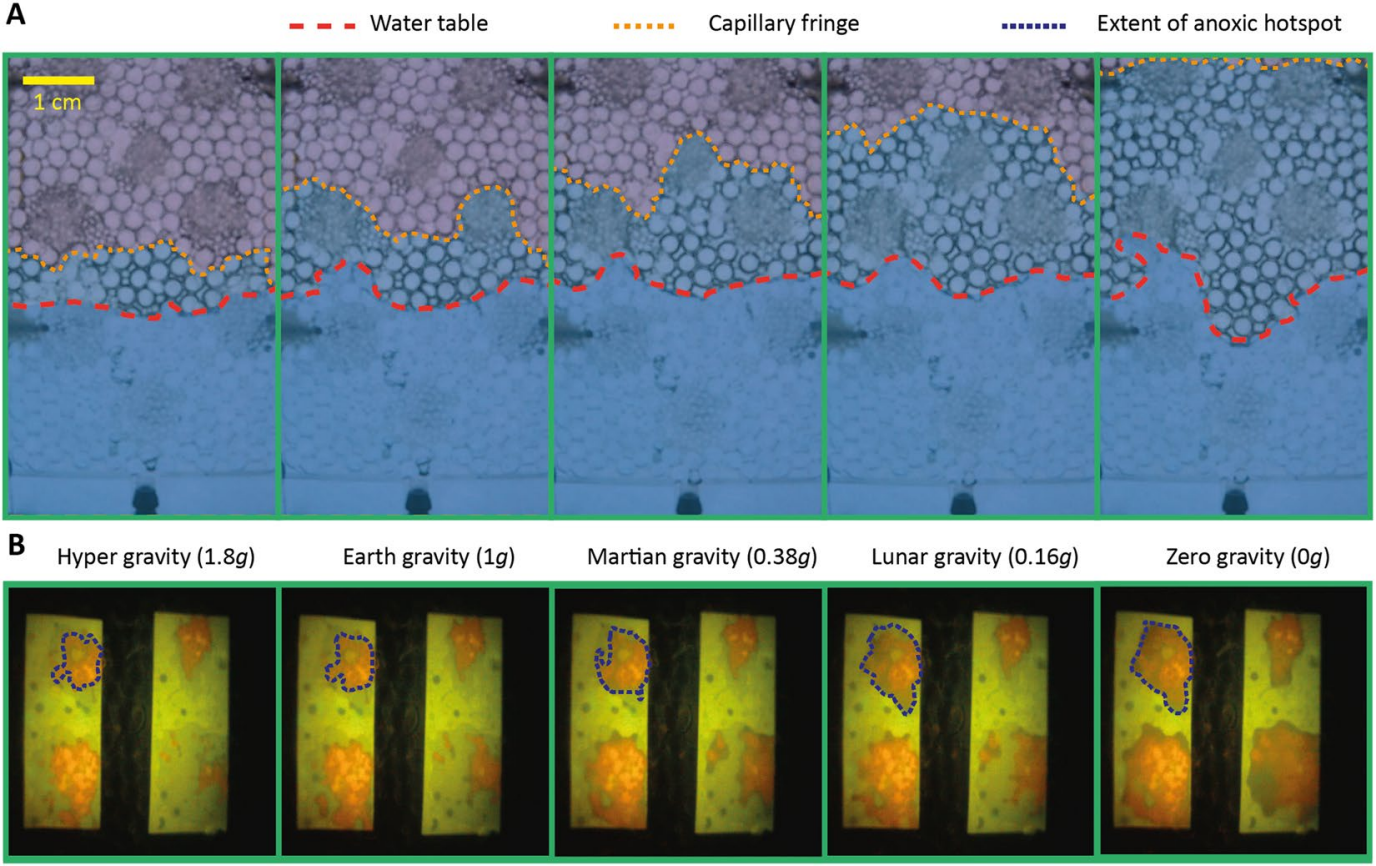
Comparison of the water table, capillary fringe and extent of anoxic hotspots depending on the gravitational conditions. (**A**) Images of the pore network showing the upper extent of the water table (red) and the capillary fringe (orange) under five different conditions of gravity. (**B**) Optode camera images showing the extent of the anoxic conditions (blue dashed line) around the upper aggregate as it changes with gravitational conditions. An increase in water availability and resulting anoxia is mainly due to liquid supply via capillary forces when less strongly opposed by gravity, and the related diffusional constraint for oxygen.

Experiments revealed that during reduced gravity, a capillary fringe extends into the previously unsaturated pore spaces, reaching and enveloping the inclusions (“aggregates”) surrounded by air under Earth gravity (Fig. 2). The extent of the capillary fringe relative to Earth conditions was dependent on gravity conditions: −0.2 cm, +0.4 cm and +1.7 cm for hyper-, Martian and Lunar gravity, respectively. During 0 *g* parabolas, the capillary fringe typically reached the top of the glass pore network (approximately +4 cm relative to Earth gravity conditions).

**Figure 3:**
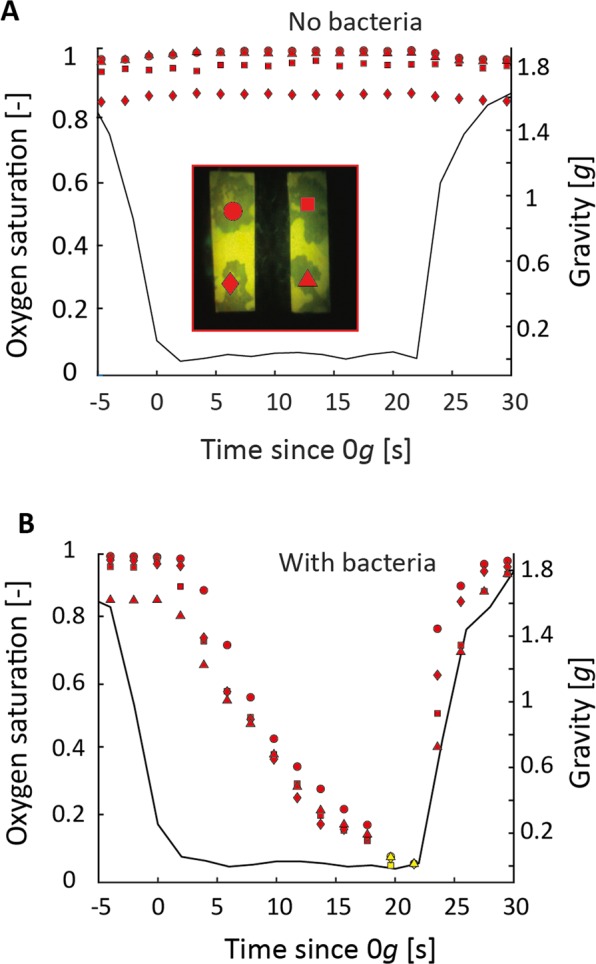
Temporal dynamics of oxygen saturation in the presence and absence of bacteria. Points show the oxygen saturation in the four upper aggregates (inset; optode foil image) during the time course of one parabola at 0 *g*, for a pore network without (**A**) and with (**B**) bacteria. The black curve depicts the gravity experienced during the same period. Oxygen saturation remains constant in the absence of bacteria, but rapid development of anoxic conditions occurs in all four hotspots in the presence of the two bacterial species. Measurements used to quantify oxygen reduction in Fig. 4 are shown by yellow markers.

**Figure 4:**
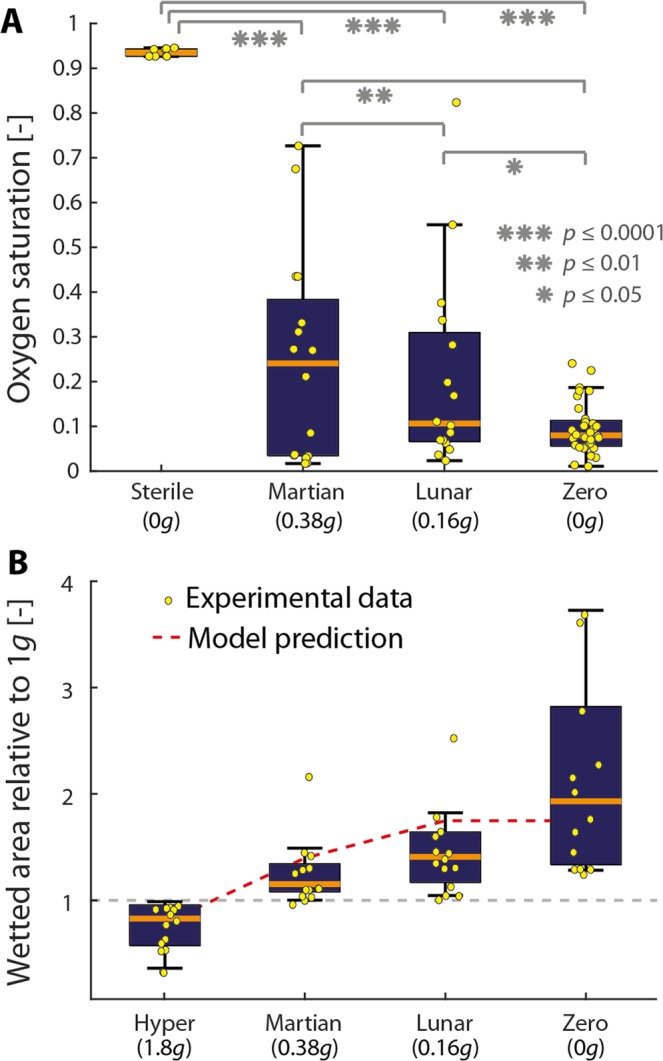
Changes in the anoxic conditions and wetted area with changing gravity conditions. (**A**) Boxplots show the minimum oxygen concentration reached at the end of the parabola (yellow data points in Fig. 3B) for three gravity conditions (Martian, Lunar, and zero gravity) and in sterile networks at zero gravity. Inoculated networks and sterile networks experiencing zero gravity differ significantly. Furthermore, a decline in residual oxygen concentration is visible with decreasing gravitational acceleration. (**B**) Boxplots show the wetted area at the end of the parabola relative to the wetted area at 1 *g* for four values of gravitational acceleration. Wetted area (the area around aggregates which are visibly wetted taken as a proxy for liquid volume around the hotspot) increases with decreasing gravitational acceleration. The geometry used in the mathematical model captures this trend well (dashed red line).

The hydrated region around hotspots formed under reduced gravity limits oxygen supply while bacteria remained active during that period consuming the remaining oxygen. In sterile networks, oxygen depletion was negligible (Fig. 3A). In contrast, presence of bacteria induced rapid oxygen depletion even during the short duration (22 s) of a single parabola (Fig. 3B). Thus, bacterial activity in the hotspots resulted in a significant reduction in oxygen saturation compared to sterile media (Fig. 4A, independent two-sample t-test between inoculated and sterile porous media after 0 *g*; *p* < 0.001). Inoculating the two bacterial species separately resulted in a significant difference in oxygen saturation, with lower saturation in media harbouring the facultative anaerobe than those containing the obligate aerobe (independent two-sample t-test; *p* = 0.032).

Differences in oxygen depletion patterns can be observed across the different gravity conditions (Fig. 4A). Although no significant difference was observed between Martian (0.38 *g*) and Lunar (0.16 *g*) gravities (paired-sample t-test; *p* = 0.39), oxygen saturation differed significantly for both Martian and Lunar gravities when compared to zero gravity conditions (paired-sample t-tests; *p* = 0.01 and *p* = 0.06, respectively). The general response is an increase in the wetted area surrounding hotspots with a decrease in gravitational acceleration (Fig. 4B). The relative wetted area, taken as the maximum area of the liquid surrounding the sub-aggregates normalized by the area at 1 *g*, increased monotonically with a decrease in gravity with a significant difference between Martian/Lunar gravity and zero gravity (paired-sample t-tests; *p* = 0.007 and *p* = 0.019, respectively). These observations reveal the important interplay between gravity, aqueous phase configuration and the development of anoxic hotspots.

**Figure 5:**
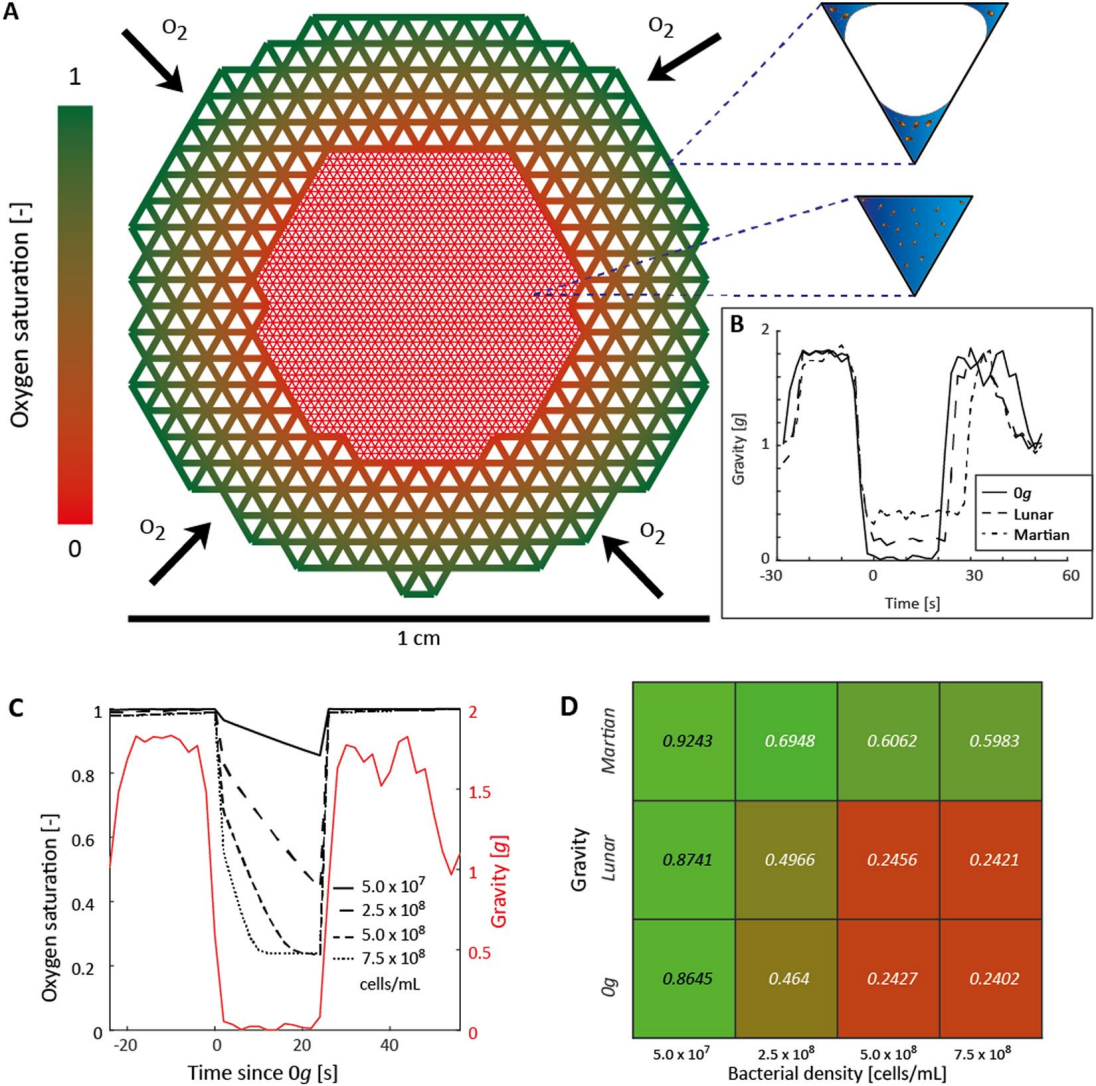
Mathematical model and simulation results investigating the effect of bacterial density on oxygen dynamics. (**A**) The mathematical model represents the porous network using an angular pore network with the inset showing a cross-section through an unsaturated and saturated triangular pore depending on pore size with water films in blue including bacterial cells. Dual porosity containing a dense inner network representing the 1-mm glass beads and coarse outer network for the 3-mm glass beads are used. The visualized oxygen profile is a consequence of bacterial consumption combined with numerical diffusion containing a constant source at the aggregate periphery. (**B**) The measured gravity dynamics were used to simulate the reconfiguration of the aqueous phase during the parabola. (**C**) A dependency of oxygen dynamics on the bacterial cell density, strategically chosen to span the observed cell densities during the experiments, is evident and shown for a zero gravity parabola. (**D**) Quantification of mean residual oxygen concentration at the end of the parabola depending on gravity condition and bacterial densities. Due to the similar predicted wetted area between Lunar and zero gravity conditions (Fig. 4B), the predicted residual oxygen concentration depending on gravity conditions for these two scenarios is very similar.

A technical constraint of inoculating the pore networks approximately 18 h before the parabolic flight restricted control of bacterial density at the time of the flight due to potential variability in overnight growth. To explore the role of bacterial density on oxygen dynamics, we thus used a mathematical model (IndiMeSH^22^) combining numerical oxygen diffusion dictated by the aqueous phase configuration with and individual based representation of bacterial growth dynamics to predict spatial oxygen concentrations within the porous media (Fig. 5A). Parameterizing the model with the accelerometer data recorded during the parabolic flights (Fig. 5B) and the experimental pore geometry yielded a good fit between the predicted and the observed distribution of the aqueous phase for all gravity conditions (Fig. 4B), providing a successful benchmark for the model. In 0 *g* conditions, model results reveal a reduction in the residual oxygen saturation at the end of a parabola with increasing bacterial density (Fig. 5C). A pattern in residual oxygen saturation can be observed when simulating the different population densities depending the gravity conditions (Fig. 5D) with more pronounced hypoxia following lower gravity conditions due to thicker water films restricting oxygen diffusion.

## Discussion

Our results from two parabolic flights show that bacterial activity in porous media under reduced gravity conditions can result in spatial distribution of anoxic zones that do not occur under Earth’s gravity. Direct, dynamic visualization of spatial oxygen concentrations using film-based optodes revealed that anoxic zones develop very rapidly, within a period of reduced gravity lasting only 22 s. The transparent porous system comprised of sintered glass beads with inclusions of smaller beads and agar mimicking bacteria-inhabited soil aggregates (“hotspots”) enabled direct visualization of aqueous phase redistribution due to a balance shift between gravitational and capillary forces and associated oxygen spatial dynamics. By systematically comparing conditions with those in sterile porous networks, we found that localized bacterial oxygen consumption induces the emergence of anoxic zones. The expansion of saturated zones around the bacterial hotspots during reduced gravity limits oxygen diffusion (due to the approximately 1000 fold lower diffusion in the aqueous compared to gas phase), hereby reducing the flux of oxygen to the hotspots such that bacterial oxygen consumption outweighs replenishment, rendering small volumes anoxic. These results were confirmed using a mathematical model, which further revealed the role of bacterial cell density on the creation of anoxic hotspots.

The experimental system and boundary conditions were engineered to enable observation of bacterially mediated oxygen consumption due to aqueous phase redistribution within the constraints of having only 22 s of zero gravity during the parabolic flights. The choice of pore network geometry and pore sizes were crucial to create a scenario where water can drain during the 1 *g* phases between parabolas while capillary forces can still restructure the aqueous phase within the timescale of one parabola. A limitation to controlling bacterial cell density during the parabolic flight experiments introduces uncertainty into the interpretation of the observations (measurements were obtained 6–8 h after the final parabola). Controlled experiments on the ground following the same inoculation procedure and measurements at the approximate times of the flight (i.e., the same delay between inoculation and the actual flight) yielded bacterial densities between 10^7^ and 10^8^ cells/mL. These values are in line with measurements in soils (e.g., 4 × 10^7^ cells/mL)^23^, suggesting that the bacterial densities in the experiments were realistic for natural conditions. Additional support for the observations was provided by model results (IndiMesh) for similar initial and boundary conditions that captures similar trends as observed during the parabolic flights.

The observed enhancement of bacterially mediated anoxic hotspots under reduced gravity has important implications for life support and other biologically mediated systems in space exploration. The occurrence of anoxia in hydrated porous media has been reported for experiments in the Space Shuttle and MIR space station^14^ and also aboard parabolic flights^15^. However, these experiments studied anoxia in the context of root respiration without explicitly considering the contribution of microbial communities. The interplay of physical, aqueous and microbial processes in soil in the context of reduced gravity has been investigated *in silico*^24^, where anaerobic metabolism (denitrification) was increased disproportionally in reduced gravity conditions. A potential ramification of such enhanced anaerobic bacterial activity is greater production and emission of greenhouse gases (40% more CO_2_ and 80% more N_2_O). Similarly, our experiments predict a higher abundance of anoxic bacterial hotspots within the pore networks, which will increase the fluxes of anaerobically produced greenhouse gases such as N_2_O or CH_4_. However, the mechanism promoting anoxic conditions or higher fluxes of greenhouse gases differ fundamentally. The *in silico* study primarily attributed the increase in nutrient fluxes to a reduction in leaching from the system with lower gravity conditions and thus overall higher nutrient availability. In our case, a reconfiguration of the aqueous phase led to more anoxic bacterial hotspots due to localized diffusion barriers surrounding the aggregates. Although this effect may seem negligible due to its spatial confinement to a small fraction of the total volume, anoxic hotspots in soil contribute disproportionally to global biogeochemical cycles. For example, 1% of the soil volume – the microbial hotspots – can account for 95% of total nitrous oxide emissions^25^ and are in turn responsible for up to 60% of the total nitrous oxide flux to the atmosphere^19^. Understanding the formation of these anoxic hotspots is therefore crucial to understand bacterial metabolism in reduced gravity conditions.

In contrast to the problems caused by anoxic bacterial metabolism, an increase in the abundance of anoxic hotspots could be beneficial in applications dependent on metabolic processes that are only possible in the absence of oxygen^26^. One example is soil remediation of perchlorates, which would be necessary on Mars before agricultural and other uses. Perchlorate concentrations of up to 0.6% by mass have been observed over wide areas on Mars^20^. On Earth, perchlorate remediation processes often include anaerobic bacterial transformation^21^. Due to the increased abundance of bacterially mediated anoxic hotspots at the gravitational acceleration of Mars, this process would be facilitated. In addition, the benefit of this process in creating a habitable environment is twofold: removal of toxic perchlorate from soil and generation of oxygen and water as by-products.

The present study revealed an increase in the abundance of bacterially mediated anoxic hotspots upon a reduction in the gravitational acceleration, due to an increase in the hydrated pore volume and the associated restrictions to gaseous diffusion. Further investigations are required to determine the effects on greenhouse gases production^24^ and rhizosphere community dynamics. Such experiments could, for instance, be achieved on board the ISS using centrifuges that enable long-term experiments at Lunar or Martian gravity (with on-board control experiments at 1 *g*)^27^. These experiments are crucial to engineer appropriate plant growth substrates to ensure reliable BRLSS and food production on future deep space exploration and colonialization of neighbouring planets.

## Methods and materials

### Parabolic flight campaign

The experiments were conducted as part of the 3^rd^ Swiss Parabolic Flight campaign over a course of two days in June 2018. Each flight consisted of 14 zero gravity parabolas, together with one Lunar and one Martian gravity parabola. Parabolas were grouped into four sets of four parabolas with a five minute break between sets, and approximately 90 s of 1 *g* between individual parabolas. Zero gravity was experienced for approximately ~22 s per parabola, slightly extended for the Lunar (~25 s) and Martian (~32 s) parabolas. Each parabola consisted of a pull-up phase (1.8 *g*), zero gravity phase (0 *g*, 0.16 *g* or 0.38 *g* depending on zero, Lunar or Martian gravity), followed by a pull-out phase (1.8 *g*), before stable flight at 1 *g*. Five different gravity conditions could therefore be investigated (1.8 *g*, 1 *g*, 0.38 *g*, 0.16 *g* and 0 *g*).

### Glass pore networks

Glass pore networks were designed to mimic the physical and chemical conditions found in soil while providing the ability to control boundary and initial conditions. A dual hierarchy of pore sizes was achieved using a monolayer of 3 ± 0.02 mm borosilicate glass beads (SiLi Beads, Sigmund Linder GmbH, Warmensteinach, Germany) in which spherical hotspots (~6 mm diameter) of 0.751 mm glass beads (SiLi Beads, Sigmund Linder GmbH, Warmensteinach, Germany) were embedded (see Fig. 1 for a conceptual representation and photo). The regions of 3 mm and 1 mm beads are associated with pore sizes of approximately 1 mm and 300 μm, respectively. The choice of pore sizes was made to ensure saturation of the small bead hotspots even under hyper gravity (1.8 *g*) during the pull-up and pull-out phases. The front cover plate of the pore networks consisted of glass with eight holes (4 mm diameter) to enhance oxygenation of the liquid phase. A PDMS membrane (125 μm thickness, SSP-M823-005, Shielding Solutions, Great Notley, UK) between the glass beads and the cover glass restricted liquid leakage whilst enabling diffusion of oxygen into the pore space. The PDMS membrane was attached to the cover glass by plasma treatment. The glass pore networks were sealed by sintering the beads and borders to the base plate and sealing of the cover plate using epoxy. Each network was connected to a liquid reservoir (an elastic balloon filled with 5 mL of bacterial growth medium encapsulated in a 3D-printed flask for stability) and a gas reservoir (a 50 mL IV bag, Easyflex+, Macopharma) using two Luer-Lock tubing adaptors (Matthes Sterilgutversorgung, Pockau-Lengefeld, Germany). Gas and liquid reservoirs were necessary to allow unconstrained movement of the liquid phase during reduced gravity.

### Pore network inoculation

In order to ensure sufficient bacterial numbers during the flight, inoculation of the glass pore networks was performed approximately 18 h prior to take off. A 40 µL aliquot of M9 minimal media agar containing 20 mM citrate was pipetted into each micro-bead hotspot inside the pore networks and immediately cooled to −20 °C for 30 s to ensure spatial confinement of the agar within the micro-bead aggregates. After solidification of the agar, 1 µL of bacterial overnight culture (diluted to OD_600_ = 0.1, corresponding to 50,000 cells) was pipetted onto the agar of each aggregate. Two motile bacterial species were chosen for this study, the obligate aerobe *Pseudomonas putida* KT2440 and the facultative anaerobic denitrifier *Pseudomonas stutzeri* A1501. These two species are commonly found in soil and the rhizosphere^28^, are able to grow in contrasting environments concerning their oxygen preference and routinely cultured in defined media. Finally, oxygen optode foils were placed onto the upper four micro-bead aggregates and the network was sealed using epoxy. After sufficient curation of the epoxy (approximately 3 h), liquid reservoirs were saturated without pressure with M9 minimal media containing 20 mM of citrate and attached to the glass pore networks. An additional 2 mL of the same growth media was added to each network, saturating approximately the lower third of the pore space. Finally, gas reservoirs were attached to the pore networks and microcosms were incubated at room temperature overnight in the dark.

### Experimental scenarios

SI Fig. 1 shows a conceptual image of the experimental scenarios tested within the four sets of four parabolas in each flight, with the same scheme replicated in the second flight (i.e., two complete biological replicates). In each flight, two optode cameras were available to monitor two individual pore networks simultaneously. Each flight consisted of sixteen parabolas split into four sets (i.e., sets of four parabolas with 90 s intermissions followed by a five-minute intermission in-between sets). The first parabola set (all 0 *g* parabolas, same experimental conditions for both optodes) consisted of pore networks containing both species representing the main experimental system. In the second set (all 0 *g* parabolas), each species was inoculated separately (i.e., pore network containing only *P. putida* imaged using one optode, pore network containing only *P. stutzeri* imaged with the second optode). In the third set (all 0 *g* parabolas), a sterile pore network containing no bacteria was monitored using one optode, and another with both species but without the addition of the carbon to the aggregates using the second optode. In the final set of four parabolas, the pore network from the first set containing the two species was reused, and exposed to one parabola at Lunar gravity, one at Martian gravity, and two parabolas at 0 *g*. In addition, this verified that there was no change in observed response during the parabolic flight when comparing the first parabola with parabola 16. For the normal gravity (1 *g*) and hyper gravity (1.8 *g*) scenarios, images during normal flight (in between parabolas) and pull-up phase were used, respectively. Integrated over the complete parabolic flight campaign (both flights), this resulted in four biological replicates (individual pore networks) of the main experimental condition with each six technical replicates (individual parabolas) for 0 *g* and one technical replicate for Lunar and Martian gravity. All other conditions (individual species, sterile, no carbon) had two biological replicates with each four technical replicates.

### Visualisation and quantification of oxygen distribution in micro-bead hotspots

Oxygen optode foils (SF-RPSu4, PreSens Precision Sensing GmbH, Regensburg, Germany) were placed across four spherical hotspots in the upper half of the network (which is drained under Earth gravity conditions) between the glass beads and the PDMS membrane. Images were taken using an oxygen optode (Detector Unit DU01 with software AnalytiCal1, PreSens Precision Sensing GmbH, Regensburg, Germany) every two seconds for the entire duration of the flight. Optode images were analysed using the KNIME software (KNIME AG, Zürich, Switzerland). After application of a Gaussian filter (σ = 2), RGB images were split into red, green and blue channels. Each hotspot was cropped to form an individual image according to manually created masks incorporating the maximal extent of the saturated area around the hotspot during the reduced gravity phase. Oxygen optodes contain two dyes: in our case, a sensitive dye emitting red fluorescence dynamically quenched by oxygen and a reference dye emitting constant green fluorescence. Oxygen concentrations can be estimated based on Fluorescence Ratiometric Imaging (FRIM) using the Stern-Vollmer relationship. This is essentially a two-point calibration taking the ratio of oxygen sensitive dye (red channel in the RGB image) to the reference dye (green channel in the RGB image) as an explanatory variable. Inability to obtain calibration images before the flight and a change of optical properties due to bacterial growth restricted the acquisition of reliable calibration images for each individual pore network. We instead calculated the change in oxygen levels during parabolas via a two-point calibration, taking the maximum R/G ratio as anoxic condition and minimum R/G ratio as oxygenated conditions. This approach could be verified by comparing calibration images (following the Presens calibration protocol) and an image of an inoculated network taken during the parabolic flight (SI Fig. 2). Due to the absence of an anoxic core in sterile networks, the mean R/G value used for the other calibrations (1.82) was used as an anoxic calibration value in this case.

### Additional data acquisition

A suite of additional parameters were obtained during the flight for verification as well as calibration purposes. Acceleration in x, y and z directions was recorded using an accelerometer (ADXL 335 5 V, Adafruit Industries, New York, USA) connected to a CR1000 data logger (Campbell Scientific Limited, Bremen, Germany). Videography of an additional pore network to directly visualise the water table and capillary fringe was performed using a GoPro camera (GoPro Hero 5 Session, GoPro, San Mateo, USA) combined with an external electroluminescence backlight panel for illumination. Capillary rise was quantified using images from the video extracted at the end of the parabola (or end of the pull-up phase for hyper gravity) in which the capillary fringe was determined manually. Image analysis software (KNIME AG, Zürich, Switzerland) was subsequently used to calculate the mean height of the capillary fringe for comparison with normal gravity conditions. Cabin pressure and temperature were obtained using two Track-It Barometric/Temperature data loggers (Monarch Instruments, Amherst, USA).

### Mathematical simulations using IndiMeSH

A previously published model (IndiMeSH) combining flux balance analysis with an individual-based representation of bacterial cells living in porous networks developed using the Matlab language (The MathWorks, Natick, MA, USA)^22^ was used to investigate the influence of cell density on the reduction in oxygenation level. A visual representation of the simulated domain is shown in Fig. 5A. Only oxygen was used as a limiting substrate, supplied from the periphery and any unsaturated nodes concerning water at a concentration of 0.27 mM (oxygen saturation at 20 °C). Matric potentials were calculated based on the elevation of the hotspot above the water table and the equation Ψ = ρ*gh*. where Ψ is the water potential [kPa], ρ the density of water [1000 kg m^−3^], *g* the gravitational acceleration [m s^−2^] and *h* the potential head [m]. Accelerometers on-board the aircraft provided measurements of gravitational acceleration. Bacterial biomass was estimated by considering individual agents with Monod growth kinetics to represent growth for simplicity. The total simulated time was 82 s (22 s plus each 30 s of hyper gravity) with 2 s time steps, covering both the hyper gravity and reduced gravity periods. The model is used to predict the residual oxygen concentration within the pore network depending on the inoculated bacterial density and oxygen diffusion restriction to the aqueous phase configuration.

## Supplementary information

Supplementary infomation.

## Data Availability

The experimental and simulation data as well as mathematical code that support the findings of this study are available from the corresponding author upon request.
