# Supplementary material for: Reduced gravity promotes bacterially mediated anoxic hotspots in unsaturated porous media
Source: Sci Rep. 2020 May 25;10:8614. doi: 10.1038/s41598-020-65362-w (PMC7248055; doi:10.1038/s41598-020-65362-w)
Supplement: Supplementary file 1 — Supplementary infomation. [file 41598_2020_65362_MOESM1_ESM.pdf]

Supplementary information

Reduced gravity promotes bacterially mediated anoxic hotspots in unsaturated porous media

Benedict Borer[1], Joaquin Jimenez-Martinez[2,3*], Roman Stocker[3] and Dani Or[1]

[1] Department of Environmental Systems Science, ETHZ, Zürich, Switzerland

[2] Department of Water Resources and Drinking Water, EAWAG, Dübendorf, Switzerland

[3] Department of Civil, Environmental and Geomatic Engineering, ETHZ, Zürich, Switzerland

*Corresponding Author: joaquin.jimenez@eawag.ch; jjiemenez@ethz.ch

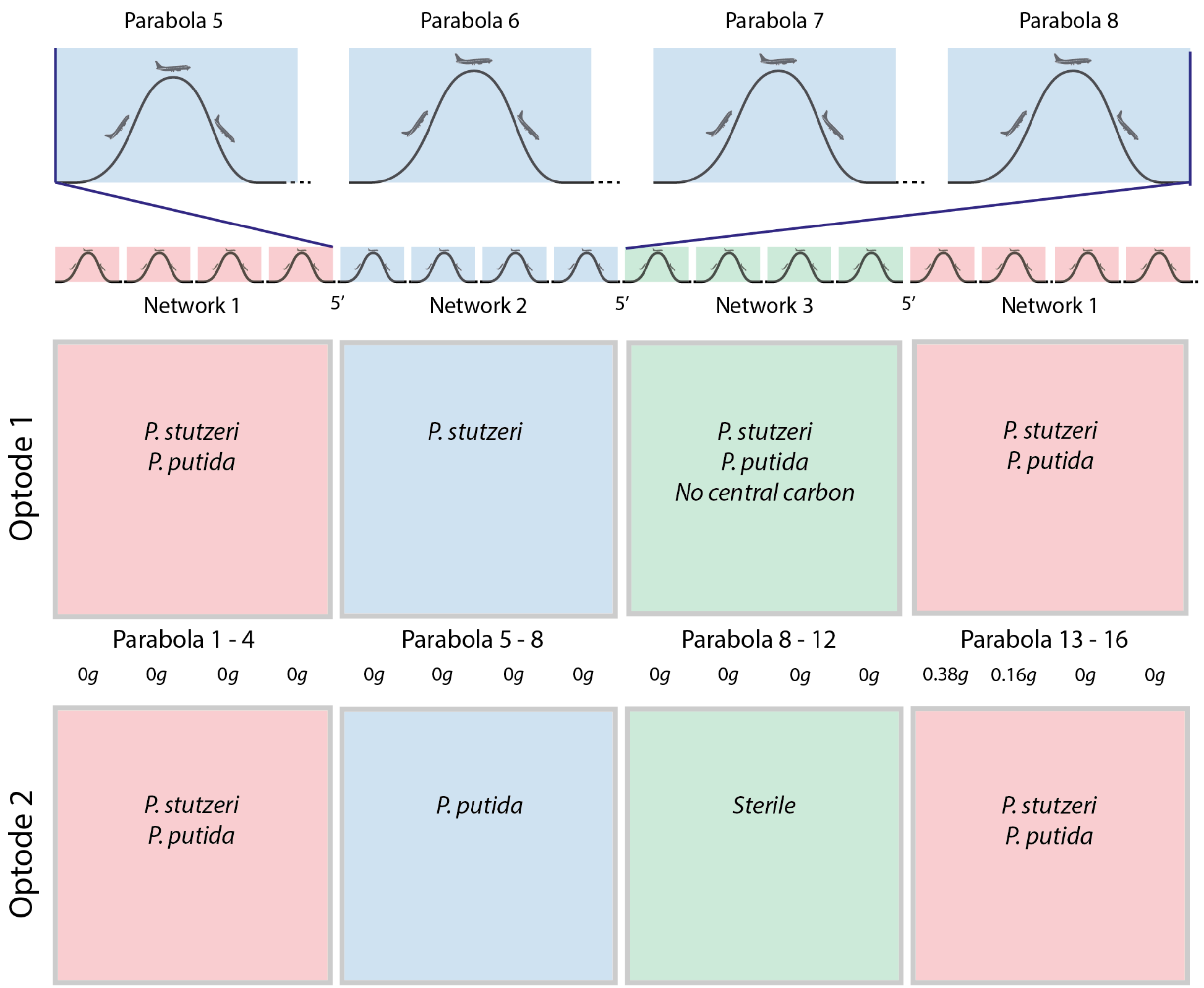

SI Figure 1: Experimental conditions within pore networks for each set of parabolas.
Each flight consisted of four sets of four parabolas, with the full scheme replicated in the second flight. All parabolas reached 0*g*, except the first two in the final set, designed to simulate Martian (0.38*g*) and Lunar (0.16*g*) gravity. Networks were only changed during the 5 min breaks between parabola sets due to time constraints. The final set of parabolas used the same pore networks as the initial set. The pore networks were inoculated with either *Pseudomonas putida*, *Pseudomonas stutzeri*, the two species together, or with no bacteria in a sterile control. In addition to studying the effect of different gravity conditions on oxygen consumption within the pore network, the set of treatments was chosen to disentangle the contribution of the two species, observe the effect of additional carbon, and compare the results to a sterile scenario.

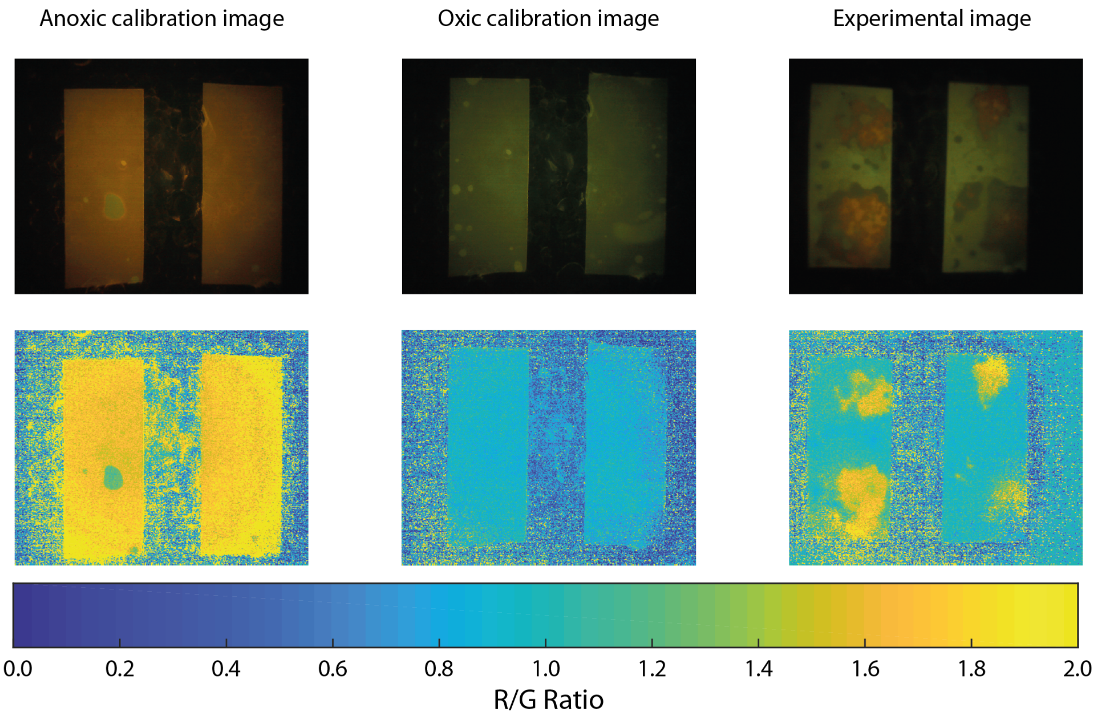

SI Figure 2: Comparison of calibration images with an inoculated network image. Photographs (top) and visualisation of the red to green pixel ratio (bottom, predictor of oxygen concentration in the Stern-Vollmer relationship as described in the Methods) for the anoxic calibration (left; sterile network filled with anaerobic liquid following the PreSens calibration protocol), oxic calibration (center; sterile network containing aerated water) and an experimental image (right). The ratio of red to green pixel intensity (color scale; used as a proxy for the level of anoxia) in anoxic and oxic calibration images are comparable to the values in the experimental image (this image was obtained during the initial second of a parabola, so the region is still oxic at the periphery). Due to the addition of agar in the centre of each hotspot and consequential saturation concerning liquid phase, this area remained anoxic throughout the flight as the high bacterial density continuously consumed diffusing oxygen. At the periphery of the hotspot, the liquid phase was either oxic at the beginning of the parabola or the optode foil was not in contact with the liquid phase. Both cases provide similar R/G ratios as shown in the oxic calibration image (the air inclusions visible on the photos as light green spots do not show up in the R/G ratio image of the oxic calibration image). Thus for each experimental image, the central anoxic region containing agar and oxic region at the periphery were used as calibration values to visualise the oxygen consumption during the microgravity phase.
